# Targeted delivery of *CEBPA*-saRNA for the treatment of pancreatic ductal adenocarcinoma by transferrin receptor aptamer decorated tetrahedral framework nucleic acid

**DOI:** 10.1186/s12951-024-02665-4

**Published:** 2024-07-04

**Authors:** Li Wang, Qunyan Yao, Xuerui Guo, Bingmei Wang, Jingyi Si, Xingye Wang, Shisong Jing, Ming Yan, Yan Shi, Guangqi Song, Xizhong Shen, Jiyu Guan, Yicheng Zhao, Changfeng Zhu

**Affiliations:** 1 Department of Gastroenterology and Hepatology, Zhongshan Hospital, Fudan University, Shanghai, China; 2 China-Japan Union Hospital of Jilin University, Changchun, China; 3 College of Clinical Medicine, Changchun University of Chinese Medicine, Changchun, China; 4 Joint Laboratory of Biomaterials and Translational Medicine, Puheng Technology, Suzhou, China; 5 Shanghai Geriatric Medical Center, Shanghai, China; 6 Department of Experimental Pharmacology and Toxicology, School of Pharmacy, Jilin University, Changchun, China; 7 State Key Laboratory of Microbial Resources, Institute of Microbiology, Chinese Academy of Sciences, Beijing, China; 8 The Third Affiliated Hospital of Changchun University of Chinese Medicine, Changchun, China; 9 State Key Laboratory for Diagnosis and Treatment of Severe Zoonotic Infectious Diseases, Key Laboratory for Zoonosis Research of the Ministry of Education, Institute of Zoonosis, and College of Veterinary Medicine, Jilin University, Changchun, China

**Keywords:** Tetrahedral framework nucleic acid, Small activating RNA, Pancreatic ductal adenocarcinoma, CCAAT/enhancer-binding protein alpha, Aptamer, RNA delivery

## Abstract

Pancreatic cancer, predominantly pancreatic ductal adenocarcinoma (PDAC), remains a highly lethal malignancy with limited therapeutic options and a dismal prognosis. By targeting the underlying molecular abnormalities responsible for PDAC development and progression, gene therapy offers a promising strategy to overcome the challenges posed by conventional radiotherapy and chemotherapy. This study sought to explore the therapeutic potential of small activating RNAs (saRNAs) specifically targeting the CCAAT/enhancer-binding protein alpha (*CEBPA*) gene in PDAC. To overcome the challenges associated with saRNA delivery, tetrahedral framework nucleic acids (tFNAs) were rationally engineered as nanocarriers. These tFNAs were further functionalized with a truncated transferrin receptor aptamer (tTR14) to enhance targeting specificity for PDAC cells. The constructed tFNA-based saRNA formulation demonstrated exceptional stability, efficient saRNA release ability, substantial cellular uptake, biocompatibility, and nontoxicity. In vitro experiments revealed successful intracellular delivery of *CEBPA*-saRNA utilizing tTR14-decorated tFNA nanocarriers, resulting in significant activation of tumor suppressor genes, namely, *CEBPA* and its downstream effector *P21*, leading to notable inhibition of PDAC cell proliferation. Moreover, in a mouse model of PDAC, the tTR14-decorated tFNA-mediated delivery of *CEBPA*-saRNA effectively upregulated the expression of the *CEBPA* and *P21* genes, consequently suppressing tumor growth. These compelling findings highlight the potential utility of saRNA delivered via a designed tFNA nanocarrier to induce the activation of tumor suppressor genes as an innovative therapeutic approach for PDAC.

**Supplementary Information:**

The online version contains supplementary material available at 10.1186/s12951-024-02665-4.

## Introduction

Pancreatic cancer is one of the most lethal malignancies globally, with pancreatic ductal adenocarcinoma (PDAC) accounting for over 85% of its incidence [1]. According to the latest data furnished by the American Cancer Society, in 2022, pancreatic cancer ranked tenth in terms of incidence within the spectrum of cancer types in the United States, but it held the third position in terms of mortality rate [2]. Despite substantial strides in diagnostic and therapeutic innovations that have led to improved outcomes for many other types of cancer, pancreatic cancer continues to exhibit escalating morbidity and rather dismal prognosis without notable amelioration [3]. Projections even indicate its ascent to become the second leading cause of cancer-related fatalities in the United States in the forthcoming years. This worrisome situation can be ascribed to the insidious onset of pancreatic cancer, which often manifests in its early stages with asymptomatic or vague symptoms, such as fatigue and anorexia. In addition, the lack of efficacious screening and early diagnostic modalities leads patients to be predominantly diagnosed at advanced stages, thereby rendering curative surgical intervention unfeasible and resulting in a 5-year survival rate less than 10%, which is incredibly low [4]. Compounding this adversity, pancreatic cancer is notorious for its profound therapeutic resistance to conventional radiotherapy and chemotherapy, which can be attributed to several pivotal features of this deadly disease, encompassing genetic instability, metabolic dysregulation, immunosuppression, hypovascularization and the presence of desmoplastic stroma [5–7]. Consequently, the majority of patients afflicted with pancreatic cancer do not benefit from emerging novel drugs and therapies, underscoring the urgent demand to explore innovative therapeutic approaches tailored to this malignancy [8]. 

While chemotherapy remains the predominant therapeutic modality for PDAC, current endeavors are increasingly directed toward modulating aberrant genetic programs [9–12]. Within this context, CCAAT/enhancer-binding protein alpha (CEBPα or CEBPA), a member of the leucine zipper-class transcription factor family, emerges prominently. This factor has been identified as a tumor suppressor, with mutations or downregulation of its encoding gene (*CEBPA*) expression recognized as pivotal contributors to oncogenesis and progression of various cancers [13–15]. Moreover, elevated expression of *CEBPA* has been demonstrated to impede tumor progression and attenuate the metastatic propensity of PDAC [16, 17]. RNA activation (RNAa) is a mechanism that enhances specific gene expression mediated by a novel category of small RNAs known as small activating RNAs (saRNAs) [18, 19]. SaRNAs can be tailed to target the promoters of tumor suppressor genes, thereby upregulating or activating their expression [20, 21]. Consequently, this process inhibits the proliferation, invasion and migration of cancer cells. Notably, saRNAs designed to target *CEBPA* have previously exhibited the capability to augment the expression of *CEBPA* along with its downstream effector, cyclin-dependent kinase inhibitor 1 (*P21*), inducing an antitumor response and diminishing tumor volume in murine PDAC models [17, 22]. These findings highlight the potential of harnessing designed saRNA-induced gene activation as a novel avenue for therapeutic intervention in PDAC.

Both saRNAs and small interfering RNAs (siRNAs) share identical chemical compositions and structures, and their differences lie in their mechanisms of action and completely opposite biological functions [23, 24]. Like siRNAs, saRNAs face the challenge of low biostability and poor cell membrane permeability when delivered to the designated location to execute their therapeutic function [25]. Over the past decade, a plethora of DNA nanostructures with well-defined shapes and sizes have been engineered as nanocarriers for nucleic acid therapeutics [26, 27]. These DNA nanocarriers, which are self-assembled from DNA molecules, offer various advantages, such as exceptional biocompatibility and biodegradability, prominent cell and tissue permeability, and high biostability. Among them, tetrahedral framework nucleic acids (tFNAs) have garnered much attention due to their facile preparation and uniformity [28]. The tFNAs have successfully delivered various nucleic acid therapeutics, including siRNAs, antisense oligonucleotides (ASOs), DNAzymes, and aptamers, for applications spanning anticancer, anti-inflammatory, antibacterial, and antiviral treatments [29–34]. 

Through abundant nucleic acid modification methods or precise base pairing, tFNAs can be easily decorated with other functional groups or molecules. For instance, aptamers, a class of functional nucleic acids obtained through in vitro selection from synthetic nucleic acid libraries, possess specific binding capabilities to receptors present on the surface of certain cancer cells [35]. These aptamers are frequently conjugated with tFNA carriers, thereby augmenting the targeting specificity and delivery efficiency of the tFNA delivery system [29, 36]. The human transferrin receptor (hTfR) is known to be overexpressed in PDAC and serves as a specific malignant marker. Therefore, targeting hTfR has been considered an effective strategy for guiding therapeutic agents to pancreatic cancer cells. Truncated transferrin receptor aptamer 14 (TR14 ST1-3 or tTR14, 22 nt) has been identified as an antagonist of hTfR and has demonstrated high internalization and affinity in PANC-1 cells [17, 37]. Herein, we developed a tTR14-decorated tFNA nanocarrier to specifically deliver exogenous saRNA targeting the *CEBPA* gene (*CEBPA*-saRNA) to pancreatic cancer cells, aiming to activate the tumor suppressor *CEBPA* and its downstream effectors to inhibit tumor cell proliferation.

## Materials and methods

### Cell lines

PANC-1 cells, a human pancreatic carcinoma cell line, were procured from the American Type Culture Collection (ATCC, Manassas, VA, USA). These cells were cultured in Dulbecco’s minimal essential medium (DMEM, Gibco) supplemented with 10% fetal bovine serum (FBS) and subsequently incubated at 37 °C with a 5% CO_2_ atmosphere.

### Preparation of tFNAs

The tFNA was prepared by mixing equimolar quantities of components S1–S4 in TM buffer (10 mM Tris base, 5 mM MgCl_2_, pH = 8.0). Next, the four single-stranded DNA samples underwent thermal treatment at 95 °C for 10 min, were rapidly cooled to 4 °C, and maintained at this temperature for 30 min [38]. To synthesize aptFNAsa, tFNA, *CEBPA*-saRNA and the tTR14 aptamer were mixed in equal ratios in TM buffer, and the final concentration of the resultant complex was adjusted to 1 µM. The mixture was then subjected to a thermal cycle of 95 °C for 10 min, followed by rapid cooling to 4 °C and stabilization at this temperature for 30 min. Cy3-tFNA, Cy3-tFNAns, and Cy3-aptFNAns were synthesized using the previously described method. Primers used for the synthesis of the respective nanomaterials are listed in Supplementary Table 2, with their detailed sequences presented in Supplementary Table 1. All oligonucleotides used in the study were synthesized and purified by Sangon Biotech Company, Shanghai, China.

### Synthetic testing of DNA nanostructures

PAGE with an 8% gel was employed to segregate biomolecules based on their electrophoretic mobility and to evaluate the successful synthesis of DNA nanostructures by analyzing the band sizes. Transmission electron microscopy (TEM) was employed to analyze the morphological characteristics of the aptFNAsa. The sample preparation followed standard protocols: nanoparticles were first prefixed with 3% glutaraldehyde, then postfixed in 1% osmium tetroxide, dehydrated in a graded series of acetone, and finally embedded. The prepared sections were examined using TEM (EP5018, Tecnai Spirit Biotwin) operating at an accelerating voltage of 120 kV. This comprehensive approach ensured detailed visualization of the nanoparticle structures, facilitating precise morphological assessments. In addition, the mean particle size and zeta potential of aptFNAsa were determined using dynamic light scattering (DLS) and phase analysis light scattering (PALS) methods, respectively, with a ZetaPALS analyzer (Brookhaven Instrument Corporation, Holtsville, NY, USA).

### Stability of DNA nanostructures at different pH values

Determination of the stability of DNA nanomaterials under different pH conditions. Briefly, 150 nM DNA nanostructures (saRNA, tFNA, tFNAsa, and aptFNAsa) were added to TM buffers at different pH values (5.0, 6.5, 7.0 and 8.2) and incubated for 1 h, followed by electrophoresis by 8% PAGE to assess the stability.

### Stability of DNA nanostructures in serum

The resilience of DNA nanostructures in serum was assessed by mixing 150 nM of each DNA nanostructure (saRNA, tFNA, tFNAsa, aptFNAsa) with cell culture medium containing 10% fetal bovine serum (FBS), followed by incubation at 37 °C. Samples were systematically collected at specific time intervals: initially at the outset (0 h) and then at 6, 12, 24, and 48 h thereafter. Subsequently, all collected samples were analyzed via 8% PAGE to assess the stability of the DNA nanostructures.

### saRNA release assay

DNA nanostructures (saRNA, tFNA, tFNAsa, and aptFNAsa) at a concentration of 150 nM were incubated with different concentrations of RNaseH (1 U/mL, 5 U/mL, 10 U/mL, or 20 U/mL) at 37 °C for 1 h. After the incubation period, 8% PAGE was utilized to assess the efficiency of saRNA release.

### Stability of DNA nanostructures in cell lysates

PAGE was employed to evaluate the stability of various DNA nanostructures (saRNA, tFNA, tFNAsa, and aptFNAsa) following incubation with PANC-1 cell lysate at a protein concentration of 500 µg/mL. Samples were collected at specific predetermined intervals: initially at time zero (0 h) and then at 6, 12, 24, and 48 h. Each sample was subsequently analyzed using 8% PAGE to meticulously assess the structural integrity of the DNA nanostructures over time.

### Confocal laser scanning microscopy

S3-Cy3 was employed for the synthesis of Cy3-tFNA, thereby enabling the evaluation of DNA nanostructure cellular uptake. PANC-1 cells were seeded into 24-well plates at a density of 5 × 10^4^ cells per well, followed by the placement of a sterile coverslip in each well and subsequent cultivation in DMEM for 24 h. Thereafter, the DMEM was removed, the wells were washed with PBS, and the medium was replaced with Opti-MEM. Cy3-tFNA, Cy3-tFNAns, and Cy3-aptFNAns, each at a final concentration of 150 nM, were incubated with PANC-1 cells at 37 °C for 2 h in a cell culture incubator. Following incubation, the cells were gently washed thrice with 0.01% PBS and then fixed with 4% paraformaldehyde for 15 min in the dark. After three subsequent washes with PBS, the cell nuclei were stained with Hoechst 33,342 (Beyotime, Beijing, China) for 10 min. The cellular uptake of the nanostructures was then observed and documented using a fluorescence microscope (Olympus, Tokyo, Japan).

### Flow cytometric analysis of cellular uptake

The cellular uptake of nanomaterials was additionally assessed using flow cytometry. In brief, PANC-1 cells were cultured at a density of 5 × 10^5^ cells per well in a 6-well plate. Following a 24-hour incubation, the Opti-MEM was replaced with DMEM, and 150 nM Cy3-tFNA, Cy3-tFNAns, and Cy3-aptFNAns were introduced to the cells for a 2-hour incubation period. Subsequently, the treated cells were harvested by trypsinization (0.25% trypsin) and collected via centrifugation. After a PBS wash, the Cy3 fluorescence intensity was measured using flow cytometry (Beckman Coulter, USA).

### Biological properties of aptFNAsa in vivo

An ectopic tumor model was established by subcutaneously injecting PANC-1 cells at a density of 1 × 10^6^ cells/0.1 mL into the subcutaneous region of five-week-old specific pathogen-free (SPF)-grade BALB/C nude mice. To assess the tumor-targeting efficacy of Cy5-labeled aptFNAsa, 100 µL of solution containing 10 µM Cy5-aptFNAsa was intravenously administered to both normal and tumor-bearing mice. Two hours postinjection, the mice were euthanized, and major organs, including the spleen, lungs, heart, liver, kidneys, and tumor, were aseptically harvested for subsequent ex vivo fluorescence imaging analysis.

### Cytotoxicity assay

The MTT assay was employed to evaluate the impact of DNA nanomaterials on the viability of PANC-1 cells. Cells were seeded into 96-well plates at a density of 5,000 cells per well and incubated for 24 h, followed by the addition of 150 nM tFNA, tFNAns, and aptFNAns. After a subsequent 48-hour incubation period, 10 µL of MTT solution (5 mg/mL) was introduced to each well. Following a 4-hour incubation, the solution was removed, and 100 µL of DMSO was added to dissolve the formazan crystals. Once the precipitate was fully dissolved, the absorbance at 570 nm was measured using a microplate reader (Multiskan FC, Thermo Fisher).

Furthermore, immunofluorescence staining was utilized to directly observe and assess the impact of DNA nanomaterials on cell proliferation and morphological alterations. PANC-1 cells were cultured in 12-well plates at a density of 3,000 cells per well and subjected to immunofluorescence staining. Alterations in cell proliferation and morphology were observed via fluorescence microscopy following the addition of aptFNAns at concentrations of 50, 100, and 150 nM for durations of 0, 24, and 48 h, respectively.

### Real-time qPCR

The RT‒qPCR assay was employed to meticulously assess the impact of nanomaterials on the transcriptional activity of the *CEBPA* and *P21* genes. PANC-1 cells were seeded into 24-well plates at a density of 1 × 10^5^ cells per well, followed by the addition of varying concentrations (50 to 150 nM) of Lipo-saRNA, tFNAsa, and aptFNAsa. The medium was refreshed every 24 h with fresh medium containing diverse concentrations of DNA nanomaterials. After a 72-hour incubation period and subsequent washing with PBS and trypsin digestion, the cells were harvested. RNA extraction was performed using a Qiagen RNeasy kit (Tiangen, Beijing, China) with DNase treatment, and the RNA concentration was ascertained using a NanoDrop 1000 spectrophotometer (Thermo Scientific). Primer sets for *CEBPa* and *p21* were designed using Primer 5, and the sequences of the forward (F) and reverse (R) primers are detailed in Table 3. cDNA amplification was conducted using Taq Pro Universal SYBR qPCR Master Mix (#R712-02, Vazyme, Nanjing, China) on an Applied Biosystems 7300 Real-Time PCR System, employing the following cycle parameters: initial denaturation at 95 °C for 30 s, followed by 40 cycles of denaturation at 95 °C for 5 seconds, annealing at 60 °C for 30 s, and extension at 72 °C for 30 s. The reference gene *18sRNA* was utilized for normalization, and the threshold cycle (Ct) values were ascertained using fixed threshold settings.

### Western blot analysis

Western blot assays were performed to analyze changes in the expression levels of CEBPA and its downstream gene P21. Briefly, PANC-1 cells were seeded into 24-well plates at a density of 1 × 10^5^ cells/well. Subsequently, Lipo-saRNA, tFNAsa, and aptFNAsa were added to the medium at a final concentration of 150 nM. PANC-1 cells without tetrahedrons were set up as a control group. The treatment was repeated every 12 h for each group, and cells were collected at 72 h. After removing the solutions and washing the cells three times with ice-cold PBS, the cells were lysed in RIPA buffer (Beyotime, China). The extracted proteins were quantified by BCA assay (Bio-Rad, Hercules, CA) according to the kit instructions. Total protein extracts were separated by SDS‒PAGE and transferred to PVDF membranes via wet transfer. After blocking with 5% skim milk, membranes were first incubated with CEBPA rabbit antibody (Cell Signaling Technology, 1:000, USA), β-actin antibody (1:1000, Proteintech, Wuhan, China), or P21 polyclonal antibody (1:1000, Proteintech, Wuhan, China) for 2 h at 4 °C. After washing the membrane with PBS, HRP-labeled goat anti-rabbit IgG (1:5000, Proteintech, Wuhan, China) was added, and the membrane was incubated at room temperature for 2 h. The protein strips were subsequently visualized with Super ECL Plus (#S6009M, US EVERBRIGHT, Suzhou, China) and visualized on a FUSION FX Spectra system (VILBER, China). The target bands were analyzed using ImageJ software (NIH).

### EdU cell proliferation assay

Cells (2 × 10^4^ cells/well) grown in 24-well plates were cultured in the presence of Lipo-saRNA, tFNAsa, and aptFNAsa for 24 h. PNAC-1 cells without any reagent were used as a control group. The effect of DNA nanomaterials on PANC-1 cell proliferation was assessed using an EdU assay kit (Beyotime, Beijing, China) according to the manufacturer’s protocol. The images were captured by a fluorescence microscope (Olympus, Japan).

### Safety assessment of aptFNAsa

All mice used in this study were five-week-old, specific pathogen-free (SPF)-grade BALB/c mice. Animals were housed in groups of five per cage, provided with ad libitum access to standard chow and water, and maintained on a 12-hour light/dark cycle. Mice were randomly assigned to one of four groups (*n* = 5 per group): a control group receiving normal saline or an experimental group receiving tFNA, tFNAsa, or aptFNAsa. Treatments were administered intravenously via the tail vein at a dose of 10 µM in a volume of 100 µL. The injections were given three times weekly for three weeks.

Throughout the 21-day experimental period, body weight was monitored continuously. At the conclusion of the study, the mice were euthanized, and serum was harvested from ocular samples. Serum samples were stored overnight at 4 °C and subsequently centrifuged at 3000 rpm for 10 min. The supernatants were collected and analyzed for a panel of hematological parameters, including alanine transaminase (ALT), aspartate transaminase (AST), total protein, albumin, and total bilirubin, as well as renal parameters, such as blood urea nitrogen (BUN).

### PNAC-1-induced pancreatic cancer mouse model

Under a controlled environment of 37 °C and 5% CO_2_, PANC-1 cells were propagated using high-glucose Dulbecco’s modified Eagle’s medium (DMEM) supplemented with 10% fetal bovine serum. During their logarithmic phase of growth, PANC-1 cells were harvested, subjected to trypsin digestion, and subsequently resuspended in PBS.

Prior to the inoculation process, sterilization was conducted in the axilla of the right forelimb of each mouse in proximity to the dorsal region. Subsequently, PANC-1 cells were subcutaneously administered into the subcutaneous region of five-week-old, specific pathogen-free (SPF)-grade BALB/C nude mice at a density of 1 × 10^6^ cells per 0.1 ml. Following inoculation, the tumors were allowed to develop over a one-week period. Thereafter, the subjects were randomly divided into four distinct groups, each receiving intravenous injections via the tail vein with PBS, tFNA (10 µM, 100 µL), tFNAsa (10 µM, 100 µL) or aptFNAsa (10 µM, 100 µL) on a schedule of three times weekly over a three-week duration. Tumor growth metrics, including changes in volume, were monitored and recorded longitudinally. Two days after the final injection, the mice were euthanized, and the tumors were excised. The weight and volume of each tumor were meticulously documented. Total RNA was isolated from the tumors of each group utilizing TRIzol reagent. This RNA was then dissolved in nuclease-free water, and its concentration and purity were verified through UV spectrophotometry. An appropriate amount of RNA was then utilized for cDNA synthesis according to the reverse transcription kit instructions. Using β-actin as an internal control, fluctuations in the transcript levels of *CEBPA* and *p21* within the tumor tissues from each group were identified. Furthermore, proteins were extracted from tumors in each group, and their concentrations were determined using the BCA method. Subsequently, fluctuations in the levels of CEBPA and P21 proteins within the tumor tissues of each group were assessed using β-actin as an internal control.

### Statistical analysis

All experiments in this study were conducted at least in triplicate. Statistical analyses were performed using the GraphPad Prism 9 software package (GraphPad Software, Inc., La Jolla, CA, USA). Quantitative data are presented as the mean ± SD. Comparisons between two groups were performed using Student’s t test, and one-way ANOVA was performed to compare multiple groups (*P* < 0.05 indicates statistical significance).

## Results

### Construction and characterization of aptFNAsa

**Fig. 1:**
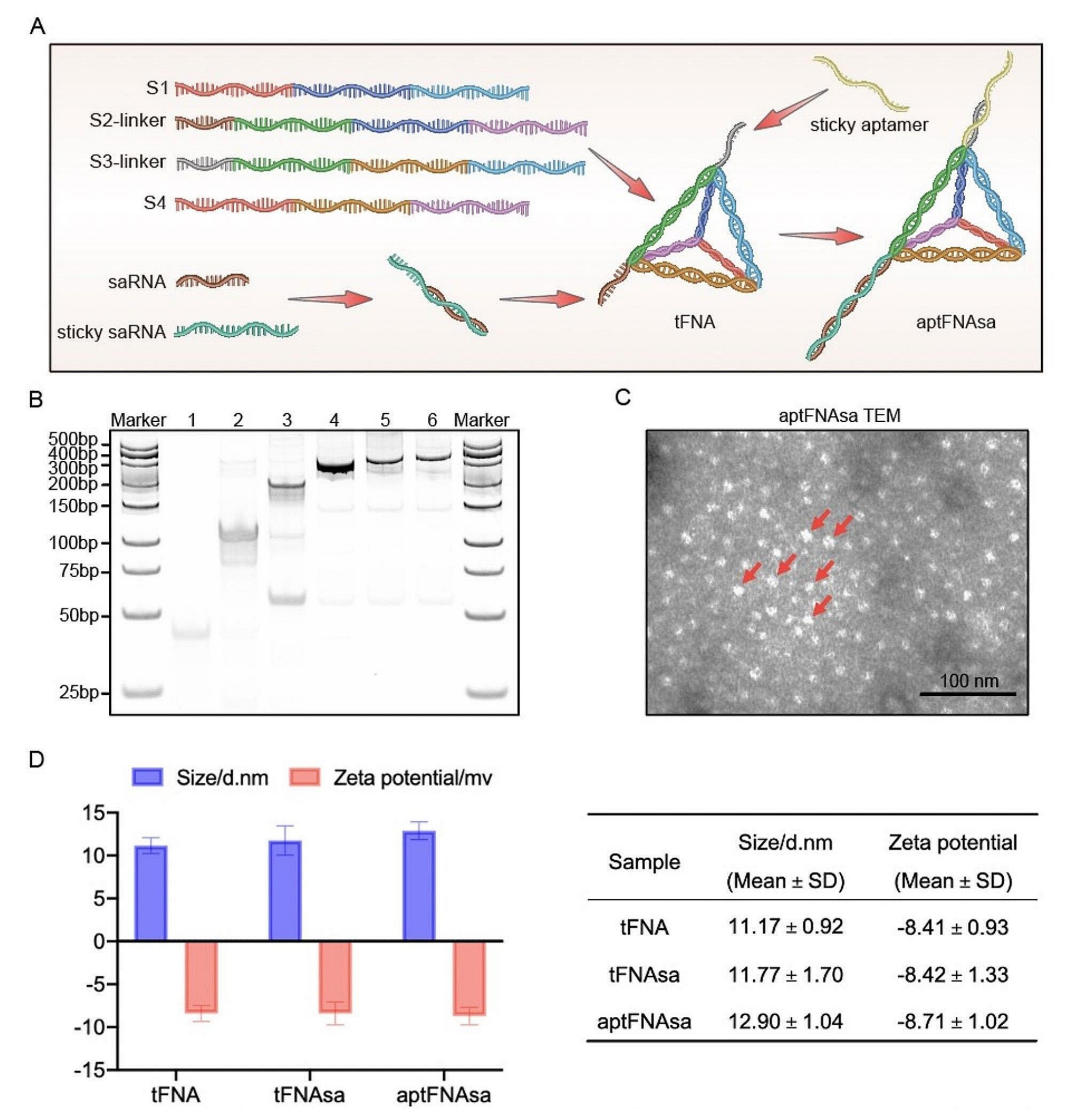
Synthesis and characterization of tFNAs. (**A**) A sketch of aptFNAsa. (**B**) Synthesis of tFNA identified by 10% PAGE. Lane 1, S1; Lane 2, S1 + S2L; Lane 3, S1 + S2L + S3L; Lane 4, S1 + S2L + S3L + S4 (tFNA); Lane 5, S1 + S2L + S3L + S4 + sticky saRNA + saRNA (tFNAsa); Lane 6, sticky tTR14 + S1 + S2L + S3L + S4 + sticky saRNA + saRNA (aptFNAsa). (**C**) Transmission electron microscopy (TEM) was used to observe the morphology of the aptFNAsa. (**D**) The apparent zeta potential of tFNA, tFNAsa, and aptFNAsa was measured using a particle size potentiometer, and the hydrated particle size of aptFNAsa was analyzed through dynamic light scattering. (F) The specific values for these measurements are presented as mean ± standard deviation (SD), providing a comprehensive statistical representation

In this study, we designed a foundational tFNA assembled from four 55-nt single-stranded DNAs (S1 ~ S4, Table S1) as building blocks. Each edge of the tFNA consisted of a 17 bp double helix, while each vertex had a 2-nt hinge (italics). In the evolutive tFNA nanocarrier, the S2L and S3L strands (Table S1) each possessed an additional 5’ sticky overhang (underscore, Table S1) protruding from the tetrahedral core. These protrusions hybridized with the corresponding stick 5’ ends (underscore, at the sense strand, Table S1) of the saRNA payload and the target recognition aptamer sequence, ultimately forming a tridimensional nanostructure decorated with the hTfR aptamer and carrying the *CEBPA*-targeted saRNA (Fig. 1A).

All nanostructures, including tFNA, tFNA‒*CEBPA* saRNA (tFNAsa), and tTR14 aptamer‒tFNA‒*CEBPA* saRNA (aptFNAsa), were simply prepared by annealing the appropriate oligonucleotides in a one-pot process (Fig. 1A and Table S2 [39]. The successful synthesis of these tFNAs was confirmed using nondenaturing PAGE, which showed distinct bands consistent with expected sizes (Fig. 1B). Transmission electron microscopy (TEM) analysis further validated the morphology and dispersion state of the aptFNAsa, indicating successful formation of the desired nanostructures (Fig. 1C). To characterize these nanostructures, we used a particle size potentiometer to determine their apparent zeta potential and dynamic light scattering to assess the hydrated particle size of aptFNAsa. The results revealed that tFNA had a size of 11.17 ± 0.92 nm, while aptFNAsa, incorporating both aptamer and saRNA, measured 12.90 ± 1.04 nm, confirming successful conjugation. Zeta potential measurements were − 8.41 ± 0.93 mV for tFNA and − 8.71 ± 1.02 mV for aptFNAsa, indicating a slight alteration in surface charge due to the conjugation (Fig. 1D, F). Overall, these results confirm the successful synthesis and characterization of the nanostructures.

### Biostability and saRNA release proficiency of engineered aptFNAsa

**Fig. 2:**
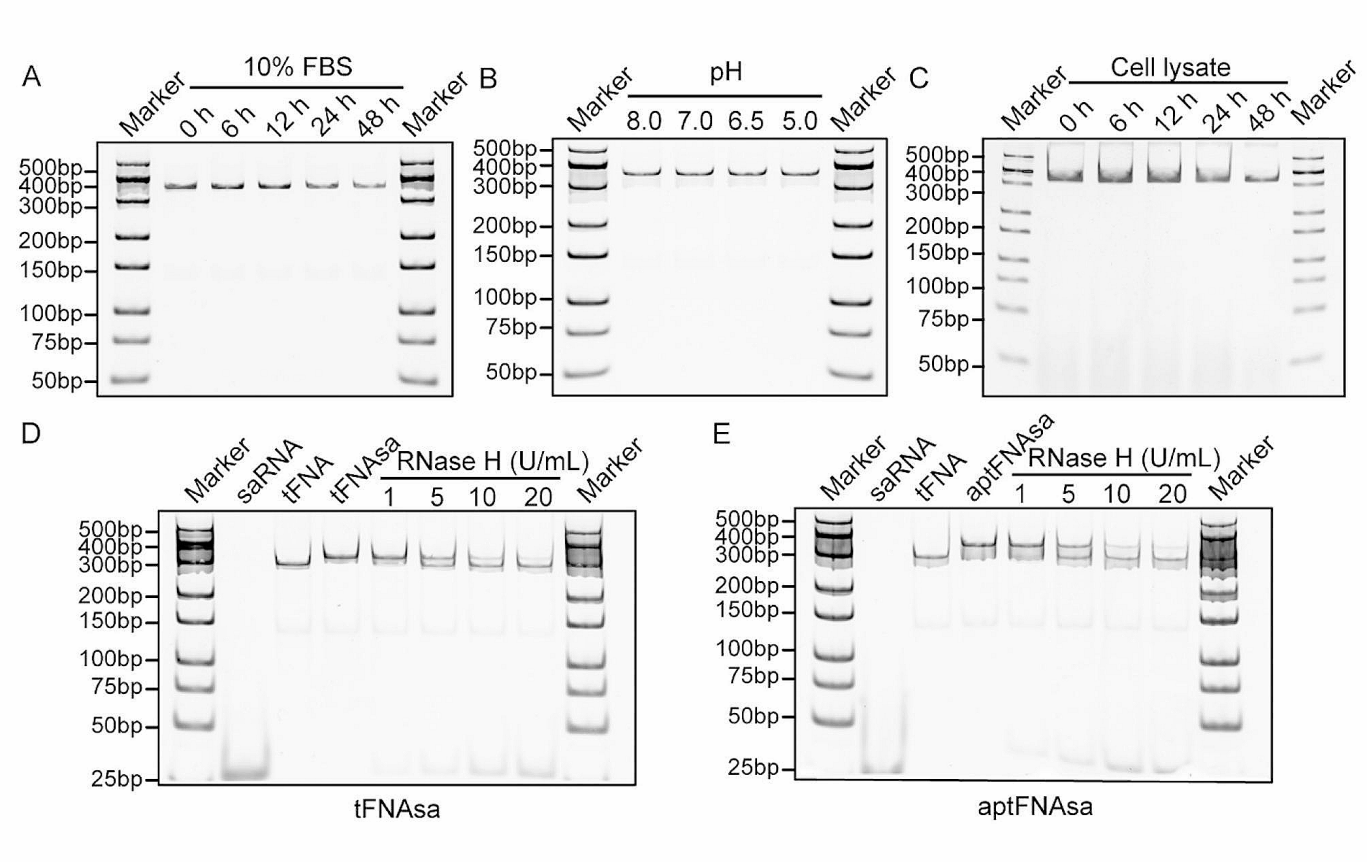
Stability assessment of aptFNAsa. (**A**) The stability of aptFNAsa incubated in 10% fetal bovine serum for 0, 6, 12, 24, and 48 h was analyzed by PAGE. (**B**) PAGE analysis of aptFNAsa was performed after incubation of samples in buffer solutions of various pH values for 1 h. (**C**) The stability of aptFNAsa incubated with PANC-1 cell lysates (protein concentration: 500 µg/mL) for 0, 6, 12, 24, and 48 h was analyzed by PAGE. (D-E) PAGE analysis of the effect of different concentrations of RNase H on the release of saRNA

The biostability of a nanostructure is pivotal for its potential medical applications. In this regard, we initiated an investigation into the stability of the engineered tFNAs within a 10% fetal bovine serum (FBS) environment. Naked saRNA underwent complete degradation following a 48-hour incubation in serum (Fig. S1A). Subsequently, we evaluated the stability of tFNAsa and aptFNAsa in serum after the incorporation of different tFNA structures. The results demonstrated that tFNAsa and aptFNAsa retained over 50% of their initial quantity even after a 48-hour serum incubation (Fig. 2A, Fig. S1B, and Fig. S1C). Additionally, we scrutinized the stability of the DNA nanostructures across varying pH conditions. As depicted in Fig. 2B and S2A-S2C, naked saRNA, tFNAsa, and aptFNAsa exhibited structural integrity, evading degradation across a range of moderately acidic to alkaline buffer environments. Subsequently, we analyzed the stability of various nanomaterials incubated with PANC-1 cell lysates (protein concentration of 500 µg/mL) for 0, 6-, 12-, 24-, or 48-hours using PAGE. The results revealed significant degradation of the standalone saRNA. In contrast, the tetrahedral complex exhibited partial stability for up to 48 h, as demonstrated in Fig. 2C and Fig. S3.

To simulate intracellular conditions and assess the saRNA release proficiency of aptFNAsa, we employed RNase H, an enzyme widely present in mammalian cells that can recognize and cleave RNA strands in almost all RNA‒DNA heteroduplexes. As illustrated in Fig. 2C and D, the release of saRNA from the tFNA nanocarriers increased progressively in tandem with rising RNase H concentrations.

Collectively, these findings underscore the impressive stability of aptFNAsa under diverse conditions with efficient saRNA release proficiency. This emphasizes the potential applicability of the tailored aptFNAsa formulation in therapeutically significant saRNA-based interventions.

### AptFNAsa exhibits exceptional uptake and biocompatibility

**Fig. 3:**
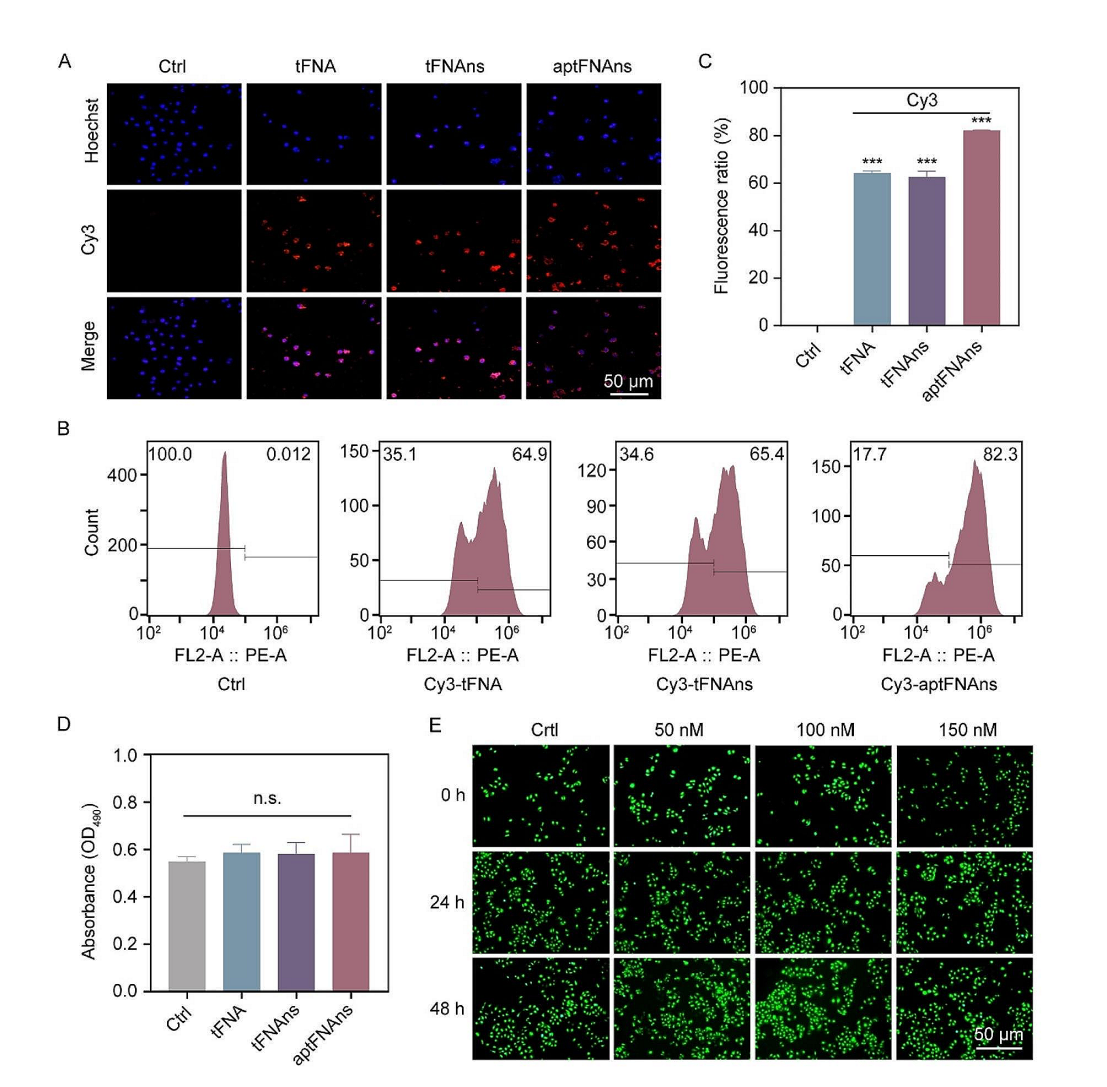
Cell uptake and biocompatibility of aptFNAns. (**A**) Laser light focusing images of cells and (**B**) flow cytometry were used to analyze aptFNAns uptake of Cy3-labeled cells. Red: Cy3-labeled tFNA; blue: Hoechst 33,342 for nuclei; Scale: 50 μm. (**C**) Quantification of flow cytometry analysis showing a fold shift in fluorescence intensity compared with untreated cells. (**D**) MTT assay was used to detect the toxicity of tFNA, tFNAns, and aptFNAns to PANC-1 cells. (**E**) Changes in cell morphology after treatment with different concentrations of aptFNAns (50–150 nM) were observed under a fluorescence microscope. Scale: 50 μm

PANC-1 cells were treated with Cy3-tFNA, Cy3-tFNA‒nonsense small RNA (Cy3-tFNAns), or tTR14 aptamer‒Cy3-tFNA‒ nonsense small RNA (Cy3-aptFNAns) for a period of 2 h, after which confocal microscopy was employed for observation. In prior investigations, it has been elucidated that both single- and double-stranded DNA molecules encounter significant barriers when traversing cellular membranes, primarily due to their intrinsic negative charge, which also predisposes them to degradation. In contrast, when DNA is structured into a three-dimensional conformation, it enables efficient cellular uptake via endocytosis. [40] The nanomaterials we have engineered, designated as aptFNAsa, feature the TR14 aptamer that specifically targets the human transferrin receptor (hTfR). Research has confirmed that the entry of the TR14 aptamer into cells is predominantly facilitated through clathrin-mediated endocytosis. [37] Our experimental results align with these previous findings. Upon the introduction of tetrahedral structures, pronounced red fluorescence was noted in the Cy3-tFNA, Cy3-tFNAns, and Cy3-aptFNAns groups (Fig. 3A). Remarkably, Cy3-aptFNAns, when paired with aptamers, demonstrated enhanced cellular uptake compared to Cy3-tFNA and Cy3-tFNAns. Furthermore, flow cytometry analysis showed that the relative fluorescence intensity of the Cy3-tFNA, Cy3-tFNAns and Cy3-aptFNAns groups were 64.23 ± 0.57%, 62.57 ± 1.42% and 82.20 ± 0.06%, respectively (Fig. 3B, C). Notably, no statistically significant difference was observed between the Cy3-tFNA and Cy3-FNAns groups (*P* > 0.05), thereby suggesting that the tTR14 aptamer was instrumental in augmenting the cellular uptake of Cy3-aptFNAns.

After our initial investigations, we assessed the cytotoxicity of PANC-1 cells in the presence of tFNA, tFNAns, and aptFNAns. Notably, at a concentration of 150 nM, these agents exhibited no discernible impact on PANC-1 cytotoxicity (Fig. 3D). Moreover, we explored the cytotoxic repercussions of tTR14 aptamer-tFNA-nsRNA (aptFNAns) on PANC-1 cells. As illustrated in Fig. 3E, PANC-1 cells were subjected to varying concentrations of aptFNAns (50, 100, 150 nM) for a duration of 48 h. Remarkably, the cell viability was analogous to that of the untreated cells, indicating no significant disparity (*P* > 0.05). This finding accentuates that the artificially engineered tetrahedral materials did not exhibit cytotoxic properties. To broaden our investigation, we assessed the potential ramifications of aptFNAns on PANC-1 cells, extending beyond cytotoxicity. Employing immunofluorescence staining, we monitored alterations in cellular morphology. Our findings disclosed that PANC-1 cells, upon interaction with aptFNAns over 24–48 h, maintained their typical cellular structure, accompanied by evident cellular proliferation over the duration.

These findings affirm the robust cellular uptake, biocompatibility, and nontoxicity of the DNA tetrahedral material, thereby paving the way for its subsequent applications.

### AptFNAsa induced tumor suppressor gene activation and antiproliferation in PANC-1 cells

**Fig. 4:**
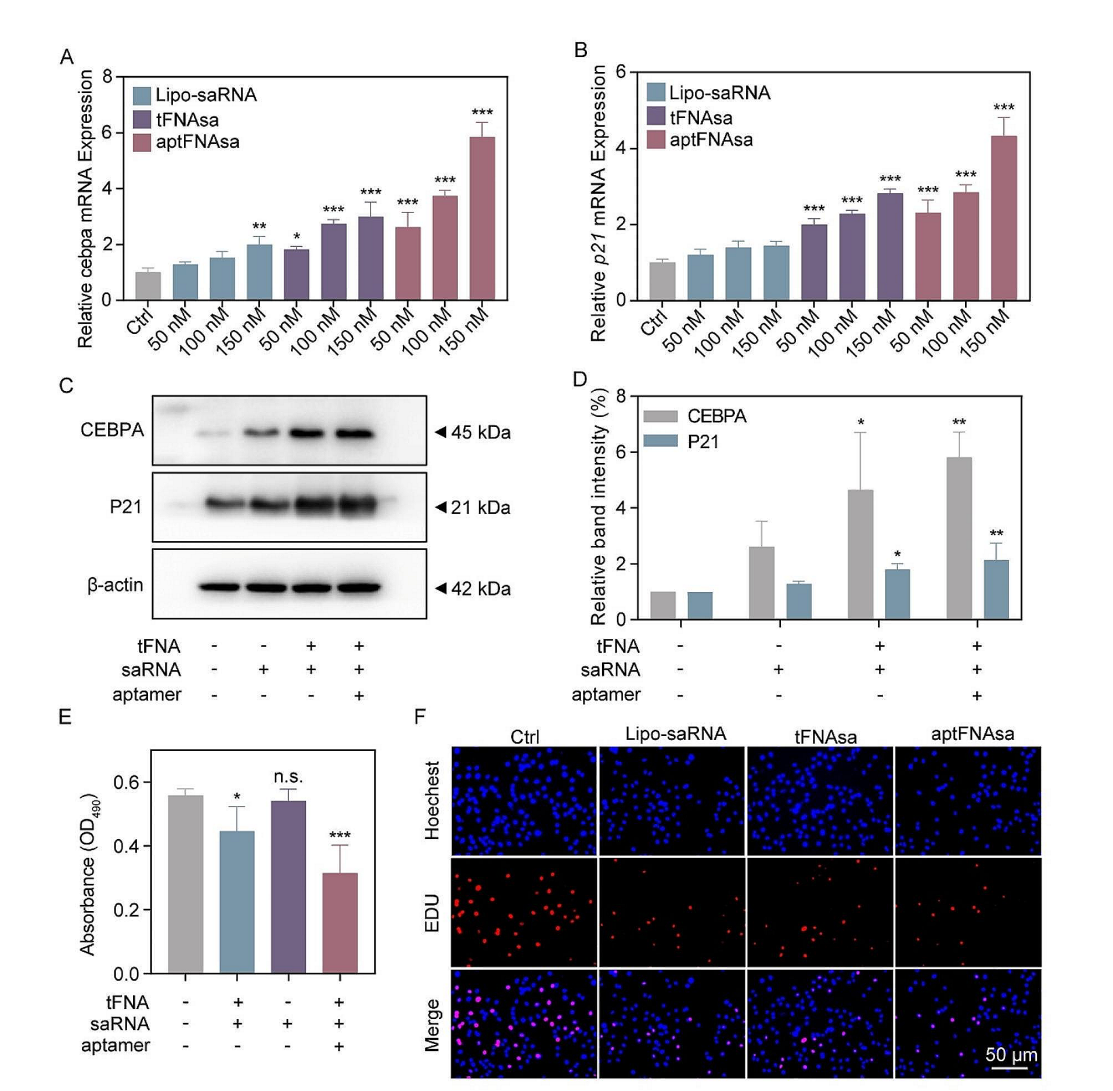
aptFNAsa upregulates the expression of CEBPA and P21 in PANC-1 cells in vitro and suppresses the proliferation of PANC-1 cells. (**A**) PANC-1 cells were treated with different concentrations (50–150 nM) of Lipo 3000-transfected saRNA (Lipo-saRNA), tFNAsa or aptFNAsa for 72 h. Untreated PANC-1 cells served as a control group. Changes in the mRNA transcript levels of *CEBPA* and (**B**) its downstream target *p21* were measured using RT‒qPCR. (**C**-**D**) Changes in CEBPA and its downstream target P21 protein levels were measured using western blot. β-Actin was used as an internal reference protein. Relative band grayscale was further assessed by ImageJ software. (**E**) Inhibition of PANC-1 cell proliferation by DNA tetrahedral material was determined using the MTT assay. (**F**) Fluorescence microscopy was used to observe the effect of DNA tetrahedral material on the proliferation of PANC-1 cells by an EdU kit. Scale: 50 μm

To evaluate the gene activation effect of saRNA by using tFNAs nanocarriers in vitro, tFNAsa or aptFNAsa was added to PANC-1 cell culture medium without any transfection reagent and cultured for 72 h. Lipofectamine 3000 (Lipo 3000) was used to transfect naked *CEBPA*-saRNA into PANC-1 cells, while untreated PANC-1 cells served as a control group. The transcription and protein levels of the saRNA-targeted gene *CEBPA* and its downstream gene *P21* were evaluated using RT‒qPCR and western blot. As shown in Fig. 4a and b, the mRNA levels of *CEBPA* and *P21* were increased by 5.86- and 4.34-fold, respectively, in aptFNAsa (150 nM)-treated cells compared to untreated cells. Furthermore, aptFNAsa-treated cells exhibited stronger activation of *CEBPA* and *P21* than tFNAsa-treated (150 nM, 3.00- and 2.82-fold compared to untreated cells) or Lipo-saRNA-transfected (150 nM, 1.99- and 1.45-fold compared to untreated cells).

Consistent results were observed in the protein levels presented by western blot analysis. Compared to the untreated group, the expression of CEBPA and P21 in the aptFNAsa (150 nM)-treated group increased by 5.82- and 2.15-fold, respectively (Fig. 4C-D). The expression of CEBPA and P21 was even greater in the aptFNAsa-treated cells than in the saRNA (150 nM, 2.61- and 1.29-fold compared to untreated cells) and tFNAsa (150 nM, 4.64- and 1.80-fold compared to untreated cells) groups. This may be attributed to the fact that the tFNA structure acts as a desirable vehicle, ensuring the stability and cellular uptake of *CEBPA*-saRNA, while the decorated TfR aptamer enhances targeting of PANC-1 cells and improves transport efficiency, playing a dual role.

High expression levels of P21, a cyclin-dependent kinase inhibitor, can induce cell cycle arrest. [41] Based on this, we evaluated the effect of each group on the proliferation of PANC-1 cells using MTT and EdU assays. As shown in Fig. 4E, the MTT results demonstrated that aptFNAsa (150 nM) significantly inhibited the proliferation of PANC-1 cells, with only half the cell survival compared to untreated cells after 72 h. Additionally, fluorescence microscopy revealed that the red fluorescence of EdU in the aptFNAsa group was significantly lower than that of the other groups, indicating a significant suppression of PANC-1 cell proliferation by aptFNAsa (Fig. 4F).

In short, the designed tFNA-borne *CEBPA*-saRNA and tTR14 aptamer played a significant role in increasing the expression of CEBPA and P21 in PANC-1 cells and suppressing their proliferation.

### Safety profile of aptFNAsa in vivo

Prior to examining the therapeutic effects, we conducted a comprehensive safety assessment of the aptFNAsa. Throughout the 21-day experimental period, the administration of tFNA, tFNAsa, and aptFNAsa did not result in significant differences in body weight compared to that of the control group receiving PBS (Figure S4A). This observation suggested that the treatments were well tolerated by the mice. Further analysis of blood chemistry parameters, including alanine aminotransferase (ALT), aspartate aminotransferase (AST), total protein, albumin, and total bilirubin, and renal parameters, such as blood urea nitrogen (BUN), revealed no significant differences between the treated groups and the untreated control group (Figure S4B). These findings indicate a favorable safety profile for aptFNAsa, demonstrating its broad biosafety profile and supporting its potential use in further therapeutic applications.

### Tumor targeting and penetration of aptFNAsa

Through subcutaneous injection of PANC-1 cells, an ectopic tumor model was established. Subsequently, we evaluated the biodistribution of Cy5-aptFNAsa (10 µM, 100 µL) in major organs and tumors of both normal and tumor-bearing mice following intravenous administration, aiming to investigate the tumor-targeting potential of Cy5-aptFNAsa. Organs, including the spleen, lungs, heart, liver, kidneys, and tumor, were harvested 2 h postinjection for ex vivo fluorescence imaging. The results showed that in normal mice, Cy5-aptFNAsa primarily accumulated in the liver and kidneys (Fig. S5A). Conversely, in tumor-bearing mice, Cy5-aptFNAsa exhibited preferential accumulation within the tumor tissue, as well as in the liver and kidneys, with the tumor being the principal site of accumulation (Fig. S5B-C). This observation confirms its ability to actively target tumors.

### In vivo antitumor effect of aptFNAsa in a mouse model of PDAC

**Fig. 5:**
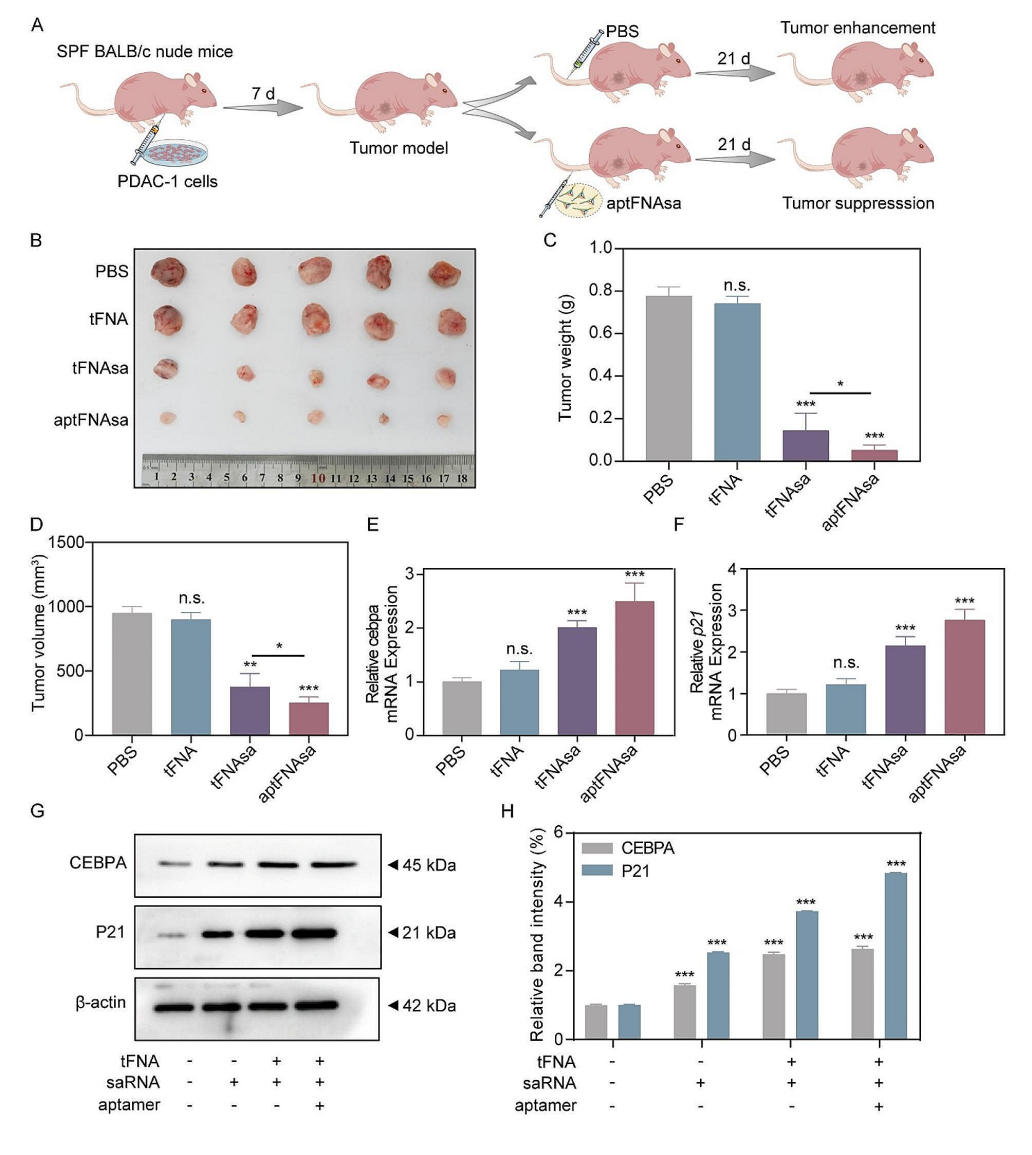
Impact of aptFNAsa on Tumor Progression in PNAC-1-Induced Murine Models. (**A**) PDAC tumor models were established in SFP-grade BALB/c nude mice via subcutaneous inoculation of PNAC-1 cells. After achieving a mean tumor volume of 150 mm³, mice were randomized into one of four groups: PBS, tFNA, tFNAsa, and aptFNAsa (*n* = 5 each). Treatments were delivered through tail vein injections at three-day intervals for a total of three weeks, with the PBS-treated group serving as the control. (**B**) Visual documentation of tumors from mice euthanized after 21 days of treatment. (**C**) Assessment of tumor weight and (**D**) volumetric analysis of tumors across all experimental groups. (**E**) RT‒qPCR-based evaluation of variations in transcript levels of *CEBPA* and (**F**) *P21*. (**G**-**H**) CEBPA and P21 protein expression levels were evaluated using western blot analysis, with β-actin serving as the loading control. The intensity of the protein bands was quantified using ImageJ software, and the relative grayscale values were analyzed

We established a xenograft tumor model via subcutaneously injecting PANC-1 cells into the axilla of nude mice, as schematically outlined in Fig. 5A. Prior to initiating treatment protocols involving aptFNAsa, preliminary data analysis revealed no notable variation in tumor dimensions across the groups. At the culmination of the 21-day observation period, the cohort treated with PBS showed a substantial increase in both mean tumor weight and volume. In contrast, mice receiving either aptFNAsa or tFNAsa exhibited a pronounced decrease in tumor dimensions and mass (Fig. 5B and D). However, the group treated exclusively with tFNA showed tumor characteristics that were statistically indistinguishable from those observed in the PBS group, confirming the potent tumor-suppressive capabilities of saRNA-directed CEBPA. Notably, aptFNAsa exhibited more significant therapeutic effects than the tFNAsa-treated group, attributed to the efficient targeting of cancer cells by the moiety in vivo. After three weeks of treatment, the mice were euthanized, and the tumor cells were harvested. After standard processing, qPCR assays were employed to probe the transcriptomic landscape of the *CEBPA* and *P21* genes. The resultant data indicated a significant increase in the transcriptional levels of both genes in the aptFNAsa-treated cohort (Fig. 5E and F). Similarly, we extracted proteins from various tumor cell groups to analyze the expression levels of CEBPA and P21. The aptFNAsa treatment group showed significant upregulation of CEBPA and P21 expression (Fig. 5G and H). These findings support the role of aptFNAsa in mitigating the proliferation of PDAC tumors in mice through the activation of the *CEBPA* and *P21* genes, demonstrating its potent in vivo antitumor activity.

## Discussion

The treatment of PDAC using conventional radiotherapy and chemotherapy is fraught with significant challenges, especially notorious resistance against conventional treatment modalities, primarily due to the inherent characteristics of this aggressive form of cancer. Overcoming these obstacles requires innovative approaches that target the underlying molecular abnormalities responsible for the development and progression of PDAC. Gene therapy has emerged as a promising strategy in this regard, offering an alternative means to address these challenges. For instance, by inhibiting oncogenes or activating tumor suppressor genes, gene therapy can effectively counteract the inherent or acquired resistance displayed by PDAC against conventional chemotherapy drugs. In particular, RNAi (RNA interference) and RNAa are two distinct mechanisms that involve the modulation of gene expression using small RNA molecules. RNAi, employing siRNAs or microRNAs (miRNAs), has been widely employed to selectively inhibit oncogenes in cancer treatment. Conversely, RNAa, while still in its early stages of research and not fully delineated in terms of its mechanism of action, has exhibited capacity for restoring tumor suppressor genes and facilitating therapeutic interventions for various cancers.

Nevertheless, the implementation of RNAa faces certain challenges that need to be addressed to optimize its efficacy. One critical consideration is the delivery of exogenous saRNAs into cells. To address this issue, we utilized tFNA, a representative tridimensional DNA nanostructure, as a nanocarrier for saRNA therapeutics in this study, aiming to enhance the delivery efficiency of saRNAs and improve their long-term effectiveness while mitigating potential off-target effects. Compared to other vectors, tFNAs offer several advantages as nanocarriers for nucleic acid therapeutics. First, the unique tridimensional structure of tFNAs provides a protective shield to nucleic acid cargos, preserving the integrity and stability of nucleic acids during circulation. Second, the compact tetrahedral shape facilitates cellular uptake through endocytosis, promoting improved delivery of nucleic acid drugs into target cells. Additionally, tFNAs are derived from nucleic acids, which are naturally occurring biomolecules, making them compatible with various physiological processes and exhibiting low immunogenicity and minimal cytotoxicity. Last, tFNAs can be easily modified to introduce additional functionalities, such as antibodies or aptamers, enabling targeted delivery to specific cells or tissues, enhancing transfection efficiency, and minimizing off-target effects. Herein, we designed a tFNA-based gene therapy formulation, aptFNAsa, by employing *CEBPA*-saRNA as a gene effector and hTfR aptamer as a targeting ligand for treating PDAC (Graphical abstract). This tailored formulation demonstrated exceptional stability, significant cellular uptake, and intracellular release of saRNA assisted by endogenous RNase H. Consequently, it led to significant activation of tumor suppressor genes, namely, *CEBPA* and its downstream effector *P21*, resulting in notable inhibition of PDAC cell proliferation in vitro and effective suppression of tumor growth in a mouse model of PDAC. It is worth noting that tFNAs provide multiple binding sites within their structures, enabling high loading capacity of multiple types of nucleic acid therapeutics simultaneously and codelivery of various therapeutic agents (e.g., small molecule drugs, nucleic acids, and proteins) to enhance their therapeutic potential.

**Scheme 1:**
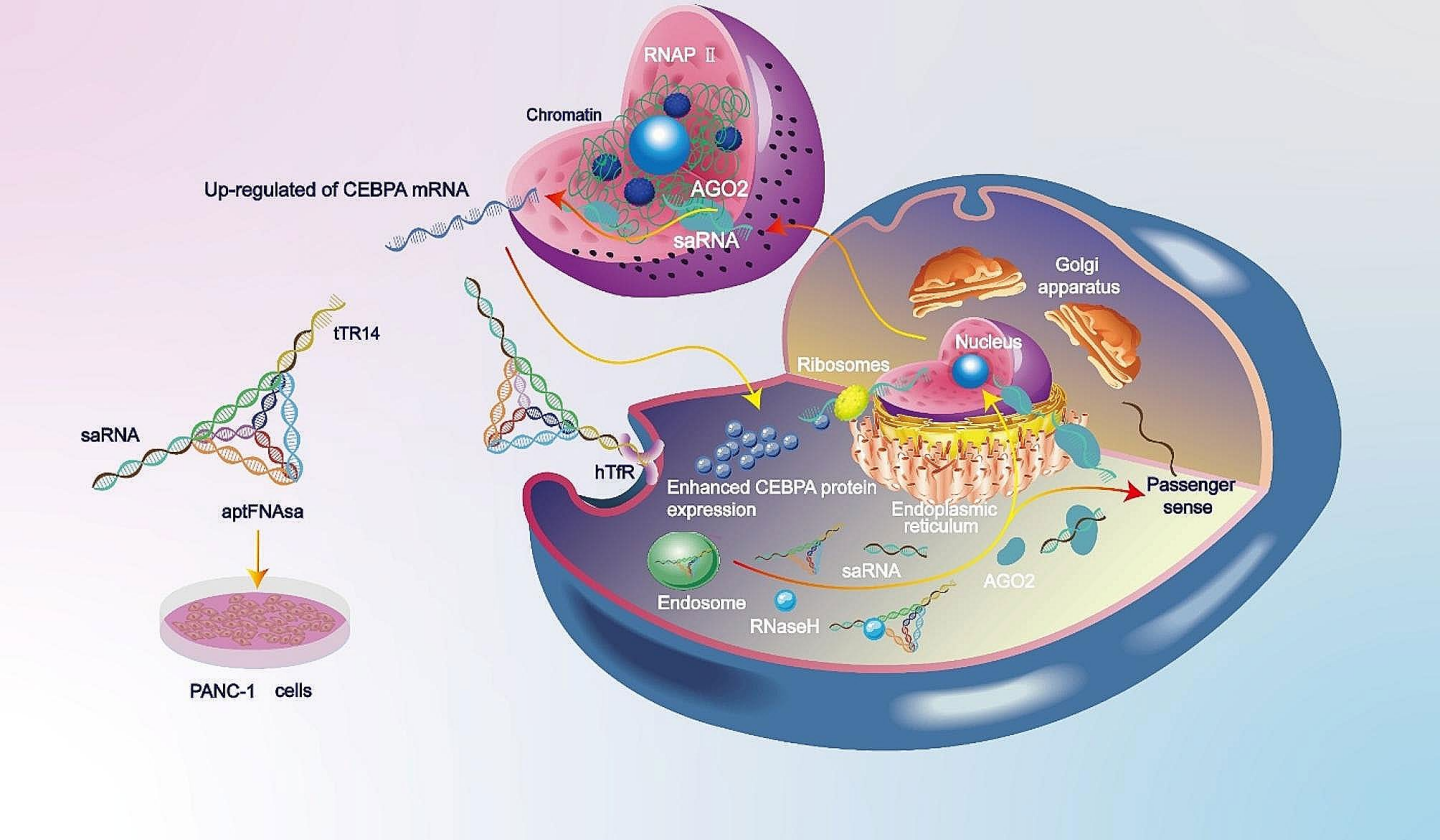
A tFNA-based gene therapy formulation employing CEBPA-saRNA and hTfR aptamer for targeted treatment of PDAC

## Conclusions

Overall, our study focuses on harnessing the potential of gene therapy, specifically RNAa, in overcoming the hurdles posed by PDAC treatment. To the best of our knowledge, this study represents the first successful utilization of DNA nanomaterials as carriers for delivering saRNA therapeutics. As illustrated in Scheme 1, by exploring the utilization of tFNA as a nanocarrier and hTfR aptamer as a targeting ligand, we successfully constructed a *CEBPA* saRNA-based formulation to activate the tumor suppressor *CEBPA* for treating this challenging form of cancer.

### Electronic supplementary material

Below is the link to the electronic supplementary material.

Supplementary Material 1

## Data Availability

No datasets were generated or analysed during the current study.

## Abbreviations

saRNA: small activating RNA
tFNA: tetrahedral framework nucleic acid
hTfR: human transferrin receptor
tTR14: truncated transferrin receptor aptamer 14 ST1-3
CEBPA: CCAAT/enhancer binding protein alpha
tFNAsa: tFNA-CEBPA-saRNA
aptFNAsa: tTR14 aptamer-tFNA-CEBPA-saRNA
nsRNA: nonsense small RNA
Cy3-tFNAns: Cy3-tFNA-nsRNA
Cy3-aptFNAns: Cy3-tTR14 aptamer-tFNA-nsRNA
ATCC: American Type Culture Collection
DMEM: Dulbecco’s minimal essential medium
FBS: fetal bovine serum
PAGE: polyacrylamide gel electrophoresis
PANC-1 cells: human pancreatic carcinoma cells
PDAC: pancreatic ductal adenocarcinoma
TEM: transmission electron microscopy
