# Supplementary material for: Targeted delivery of CEBPA-saRNA for the treatment of pancreatic ductal adenocarcinoma by transferrin receptor aptamer decorated tetrahedral framework nucleic acid
Source: J Nanobiotechnology. 2024 Jul 4;22:392. doi: 10.1186/s12951-024-02665-4 (PMC11223357; doi:10.1186/s12951-024-02665-4)
Supplement: Supplementary file 1 — Supplementary Material 1 [file 12951_2024_2665_MOESM1_ESM.pdf]

Supporting Information

Targeted Delivery of *CEBPA*-saRNA for the Treatment of Pancreatic Ductal Adenocarcinoma by Transferrin Receptor Aptamer Decorated Tetrahedral Framework Nucleic Acid

Li Wang[‡], Qunyan Yao[‡], Xuerui Guo[‡], Bingmei Wang, Jingyi Si, Xingye Wang, Shisong Jing, Ming Yan, Yan Shi, Guangqi Song, Xizhong Shen, Jiyu Guan[*], Yicheng Zhao[*], Changfeng Zhu[*]

[*] Correspondence: jiygua@jlu.edu.cn; yichengzhao@live.cn; zhuchangfeng@fudan.edu.cn

Note: Correspondence and post-publication inquiries, especially regarding Methodology and Materials, will be handled by Changfeng Zhu (email: zhuchangfeng@fudan.edu.cn)

[‡] Li Wang, Qunyan Yao, and Xuerui Guo contributed equally to this work.

Table S1. Oligonucleotides used in this paper for the synthesis of tFNA materials.

Name	Sequence (5'-3')
S1	ACATTCCTAAGTCTGAA*AC*ATTACAGCTTGCTACAC*GA*GAAGAGCC<br>GCCATAGTA
S2	TATCACCAGGCAGTTGA*CA*GTGTAGCAAGCTGTAAT*AG*ATGCGAGG<br>GTCCAATAC
S3	TCAACTGCCTGGTGATA*AA*ACGACACTACGTGGGAA*TC*TACTATGG<br>CGGCTCTTC
S4	TTCAGACTTAGGAATGT*GC*TTCCCACGTAGTGTCGT*TT*GTATTGGAC<br>CCTCGCAT
S1-Cy3	Cy3-<br>ACATTCCTAAGTCTGAAACATTACAGCTTGCTACACGAGAAGAGCC<br>GCCATAGTA
S2L	AATTGACCTGTGAATTTATCACCAGGCAGTTGACAGTGTAGCAAGC<br>TGTAATAGATGCGAGGGTCCAATAC
S3L	GGCTATAGCACATGGGTAAATCAACTGCCTGGTGATAAAACGACAC<br>TACGTGGGAATCTACTATGGCGGCTCTTC
sticky saRNA	UUCACAGGUCAAUUGCGGUCAUUGUCACUGGUCUU
saRNA	GACCAGUGACAAUGACCGCUU
sticky tTR14	TTTACCCATGTGCTATAGCCUUUAUUCACAUUUUUGAAUUGA
nsRNA	CCAUGUGAUUUUGUUGUUAAU
sticky nsRNA	UUCACAGGUCAAUUAUUAACAACAAAAUCACAUGG

Note：Underscore indicates the sticky sequences. Italic indicates the hinge bases.

Table S2. Oligonucleotide composition for different tFNAs.

tFNA types	Names of Oligonucleotides						
tFNA	S1	S2L	S3L	S4			
tFNAsa	S1	S2L	S3L	S4	sticky saRNA	saRNA	
aptFNAsa	S1	S2L	S3L	S4	sticky saRNA	saRNA	sticky tTR14
Cy3-tFNA	S1-Cy3	S2L	S3L	S4			
Cy3-tFNAns	S1-Cy3	S2L	S3L	S4	sticky nsRNA	nsRNA	
Cy3-aptFNAns	S1-Cy3	S2L	S3L	S4	sticky nsRNA	nsRNA	sticky tTR14
Cy5-aptFNAsa	S1-Cy5	S2L	S3L	S4	sticky saRNA	saRNA	sticky tTR14

Table S3. Primers used for RT–qPCR.

Name	Sequence (5’-3’)
18 sRNA-F	GTAGTCATATGCTTGTCTC
18 sRNA-R	ATTCCCCGTTACCCGTTG
CEBPA-F	CACCGCTCCAATGCCTAC
CEBPA-R	CCCATCGCAGTGAGTTCCG
p21-F	GTGGGAATTCGCATATGTCAGAACCGGCTGGG
p21-R	ACGAGGATCCAAGCTTTAGGGCTTCCTCTTGGA

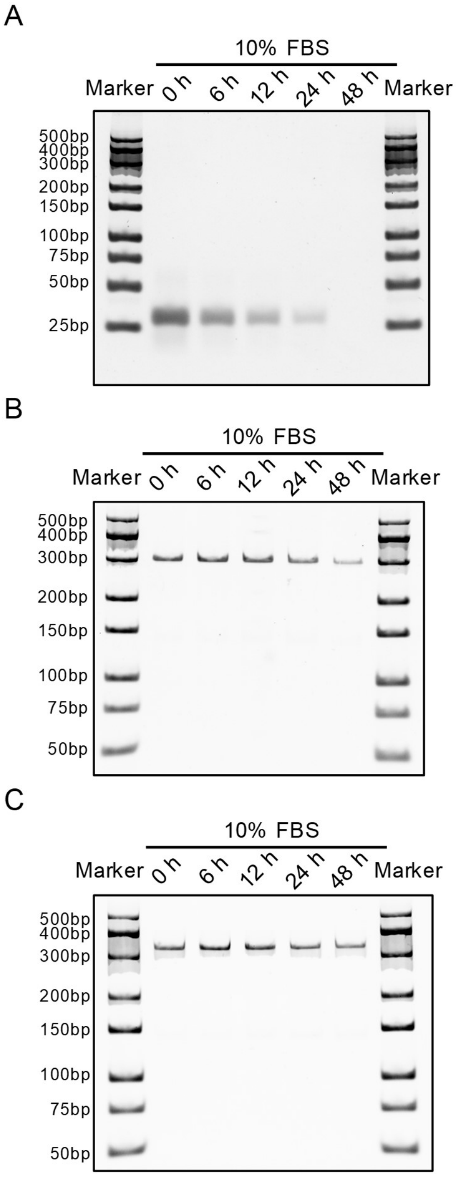

Figure S1. Stability assay of tFNAs in fetal bovine serum. (A) saRNA. (B) tFNA. (C) tFNAsa.

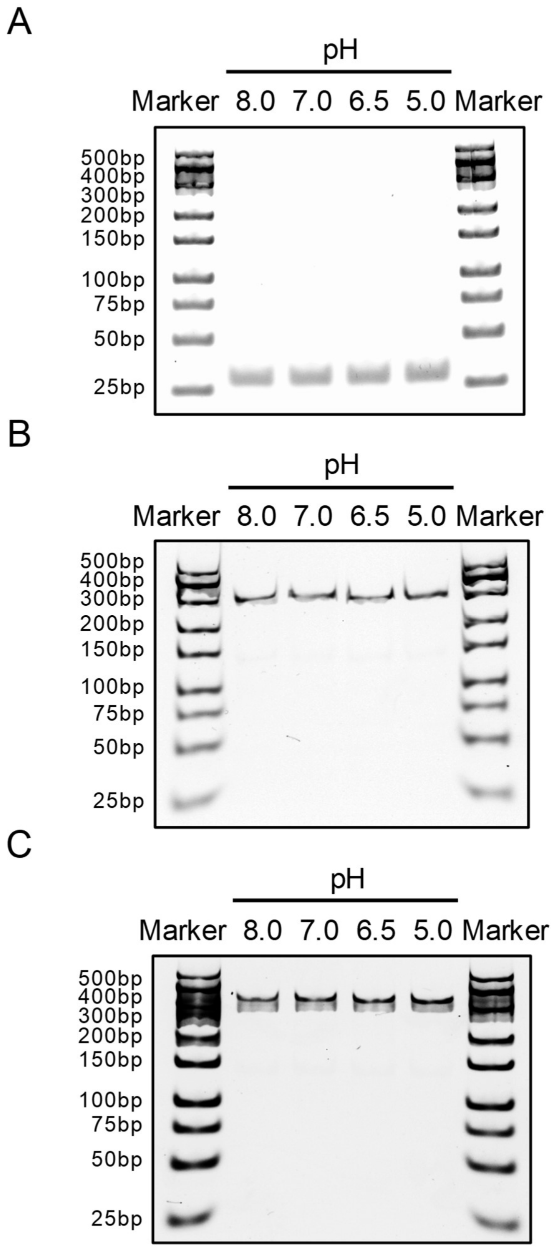

Figure S2. Effect of pH value on the stability of tetrahedral materials. (A) saRNA. (B) tFNA. (C) tFNAsa.

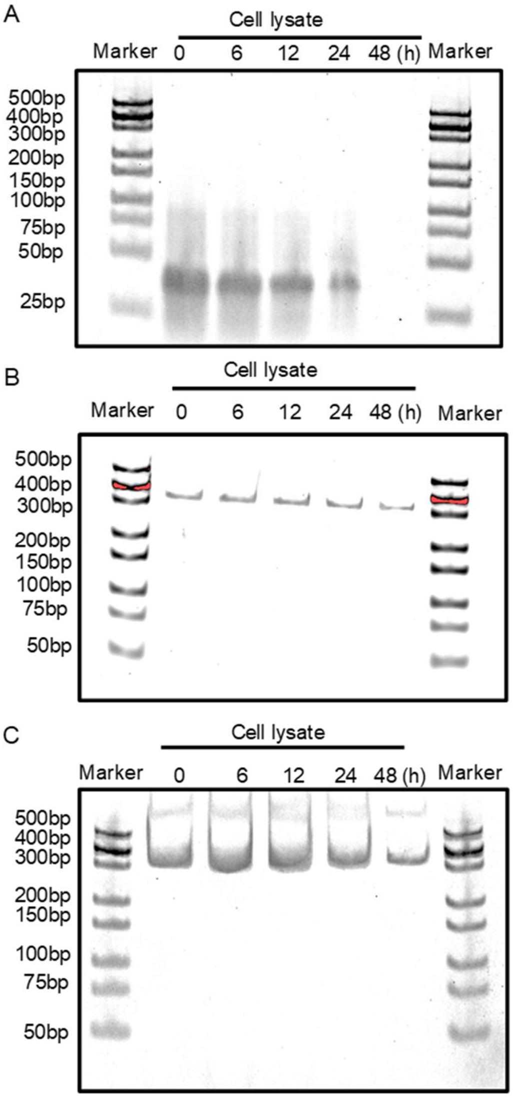

Figure S3. Effect of PANC-1 cell lysates (protein concentration:500 μg/mL) on the stability of tetrahedral materials. (A) saRNA. (B) tFNA. (C) tFNAsa.

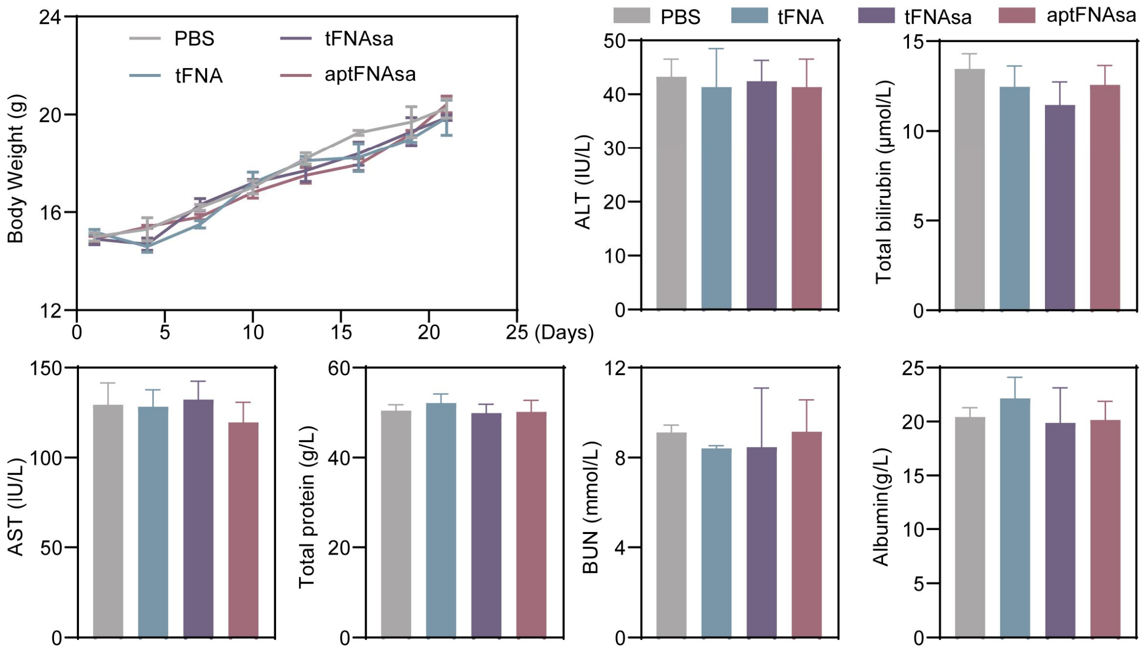

Figure S4. Evaluation of aptFNAsa biosafety *in vivo*. (A) Changes in body weight percentage in animals following intravenous administration of tFNA, tFNAsa, and aptFNAsa over a period of 21 days. (B) Blood chemistry profiles for hematological (ALT, total bilirubin, AST, total protein, albumin) and renal (BUN) parameters after treatment with aptFNAsa, indicating no significant adverse impacts on liver or kidney functions.

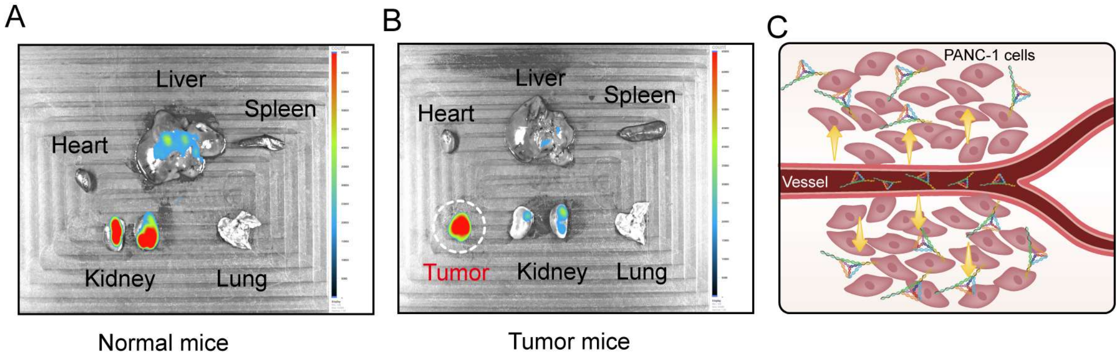

Figure S5. Biological properties of the aptFNAsa *in vivo*. (A) *Ex vivo* fluorescence imaging of key organs and tumors harvested from both normal and (B) tumor-bearing mice was conducted 2 hours post-injection to assess biodistribution. (C) A schematic diagram illustrating the active tumor-targeting mechanism of aptFNAsa.
